# Supplementary material for: Mutations in PCYT2 disrupt etherlipid biosynthesis and cause a complex hereditary spastic paraplegia
Source: Brain. 2019 Oct 22;142(11):3382–97. doi: 10.1093/brain/awz291 (PMC6821184; doi:10.1093/brain/awz291)
Supplement: awz291_Supplementary_Data [file awz291_supplementary_data.zip › awz291-Suppl_data/Supplementary_Data4.zip!/Supplementary information 3 - fibroblast lipidomics.zip!/Fibroblast lipidomics/Controle vs Patient2/Statistical plots and data/PC[O] species/Boxplots.pdf]

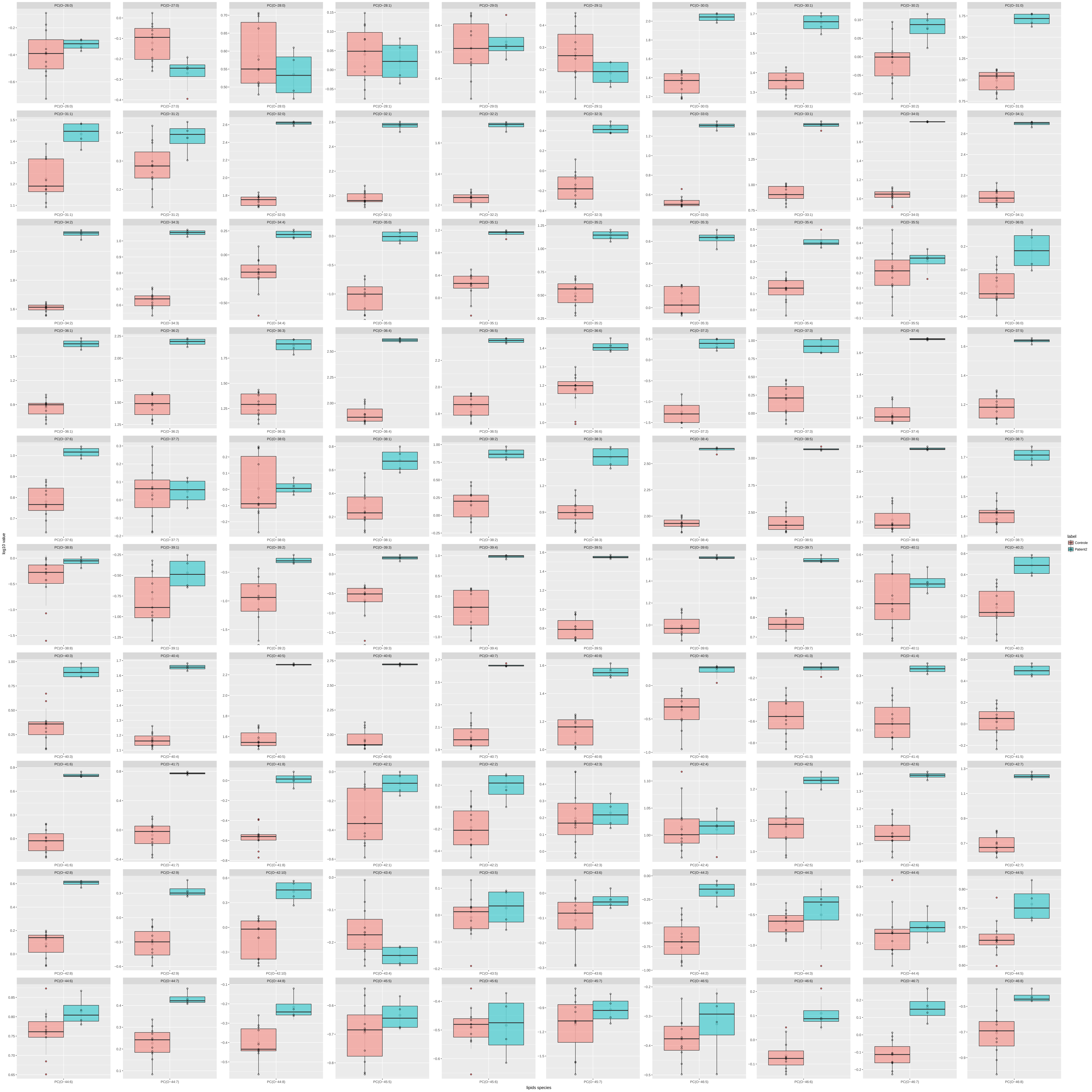
PC(O-26:0)
PC(O-27:0)
PC(O-28:0)
PC(O-28:1)
PC(O-29:0)
PC(O-29:1)
PC(O-30:0)
PC(O-30:1)
PC(O-30:2)
PC(O-31:0)
PC(O-31:1)
PC(O-31:2)
PC(O-32:0)
PC(O-32:1)
PC(O-32:2)
PC(O-32:3)
PC(O-33:0)
PC(O-33:1)
PC(O-34:0)
PC(O-34:1)
PC(O-34:2)
PC(O-34:3)
PC(O-34:4)
PC(O-35:0)
PC(O-35:1)
PC(O-35:2)
PC(O-35:3)
PC(O-35:4)
PC(O-35:5)
PC(O-36:0)
PC(O-36:1)
PC(O-36:2)
PC(O-36:3)
PC(O-36:4)
PC(O-36:5)
PC(O-36:6)
PC(O-37:2)
PC(O-37:3)
PC(O-37:4)
PC(O-37:5)
PC(O-37:6)
PC(O-37:7)
PC(O-38:0)
PC(O-38:1)
PC(O-38:2)
PC(O-38:3)
PC(O-38:4)
PC(O-38:5)
PC(O-38:6)
PC(O-38:7)
PC(O-38:8)
PC(O-39:1)
PC(O-39:2)
PC(O-39:3)
PC(O-39:4)
PC(O-39:5)
PC(O-39:6)
PC(O-39:7)
PC(O-40:1)
PC(O-40:2)
PC(O-40:3)
PC(O-40:4)
PC(O-40:5)
PC(O-40:6)
PC(O-40:7)
PC(O-40:8)
PC(O-40:9)
PC(O-41:3)
PC(O-41:4)
PC(O-41:5)
PC(O-41:6)
PC(O-41:7)
PC(O-41:8)
PC(O-42:1)
PC(O-42:2)
PC(O-42:3)
PC(O-42:4)
PC(O-42:5)
PC(O-42:6)
PC(O-42:7)
PC(O-42:8)
PC(O-42:9)
PC(O-42:10)
PC(O-43:4)
PC(O-43:5)
PC(O-43:6)
PC(O-44:2)
PC(O-44:3)
PC(O-44:4)
PC(O-44:5)
PC(O-44:6)
PC(O-44:7)
PC(O-44:8)
PC(O-45:5)
PC(O-45:6)
PC(O-45:7)
PC(O-46:5)
PC(O-46:6)
PC(O-46:7)
PC(O-46:8)
log10 value
lipids species
label
Controle
Patient2
